# Results from Brazil’s 2022 Report Card on Physical Activity for Children and Adolescents

**DOI:** 10.3390/ijerph191610256

**Published:** 2022-08-18

**Authors:** Diego Augusto Santos Silva, Andreia Pelegrini, Diego Giulliano Destro Christofaro, Elisa Pinheiro Ferrari, Gerson Ferrari, Kelly Samara Silva, Luiz Rodrigo Augustemak de Lima, Nelson Nardo Júnior, Roberto Jerônimo dos Santos Silva, Valter Cordeiro Barbosa Filho

**Affiliations:** 1 Universidade Federal de Santa Catarina, Florianópolis 88040-900, Brazil; 2 Universidade do Estado de Santa Catarina, Florianópolis 88037-100, Brazil; 3 Universidade Estadual Paulista, Presidente Prudente 18618-689, Brazil; 4 Universidade Católica de Brasília, Brasília 71966-700, Brazil; 5 Escuela de Ciencias de la Actividad Física, el Deporte y la Salud, Universidad de Santiago de Chile (USACH), Santiago 9170022, Chile; 6 Grupo de Estudio en Educación, Actividad Física y Salud (GEEAFyS), Laboratorio de Rendimento Humano, Universidad Católica del Maule, Talca 3460000, Chile; 7 Universidade Federal de Alagoas, Maceió 57051-090, Brazil; 8 Universidade Estadual de Maringá, Maringá 87020-900, Brazil; 9 Universidade Federal de Sergipe, São Cristovão 30130-003, Brazil; 10 Instituto Federal do Ceará, Fortaleza 61939-140, Brazil

**Keywords:** child health, adolescent health, health policy, sedentary behavior

## Abstract

This research aims to summarize the process and results of the 2022 Report Card on Physical Activity for Brazilian children and adolescents. A group of experts led by 10 PhD researchers gathered the best possible evidence on physical activity indicators. The Report Card Brazil 2022 included the top 10 indicators of physical activity and sleep, obesity, and poor mental health variables, which made up four dimensions: (I) Daily Behaviors; (II) Settings and Sources of Influence; (III) Government Strategies and Investments; and (IV) Health Outcomes. Comprehensive searches, including peer-reviewed and gray literature searches, were performed for each indicator. Data were considered from systematic reviews, local and national surveys, websites, and official information from the Brazilian Federal Government. Grades from the indicators ranged from F (Active Play) to B (School). In addition, the results found for each indicator were Overall Physical Activity (D), Organized Sport Participation (C−), Active Transportation (C), Sedentary Behaviors (D), Sleep (C), Family and Peers (C−), Community and Environment (C), Government (D+), Physical Fitness (D+), Obesity (11.7%), and Poor Mental Health (37.8%). Successfully strategies for increasing physical activity among Brazilian children and adolescents should look at the different indicators presented in this report.

## 1. Introduction

In 2021, the Brazilian federal government launched the “Physical Activity Guide for the Brazilian population” guideline [1]. The data reported in the Brazilian guideline come from the pre-pandemic period [1]. This document systematizes, in a single file, conceptual information on the subject through easy-to-understand language, which is to be disseminated throughout the country. Regarding recommendations for school-aged children and adolescents, the Brazilian document corroborates the recommendations of the World Health Organization [2], according to which this population subgroup should accumulate at least 60 min of moderate-to-vigorous-intensity physical activities daily; and for at least three days a week, activities to strengthen muscles and bones should be included, involving movements such as jumping, pulling, or pushing [1,2].

The recommendations for school-aged children and adolescents are based on evidence that confirms the benefits of physical activity for cardiovascular, cognitive, and mental health [3], with additional benefits being obtained if children and adolescents reduce sedentary behavior and increase levels of physical activity [1,2,3]. Given this available evidence, the Report Card in Brazil is linked with the Global Matrix project (GMp), led by the Active Healthy Kids Global Alliance (AHKGA) which seeks to monitor and summarize the best possible evidence on this topic among the Brazilian population [4,5,6]. The first edition of the Brazilian project was published in 2016 and showed that 41.7% of Brazilian school-aged children and adolescents met physical activity guidelines [7]. This prevalence declined to 31.1% in the second edition of the project, which was launched in 2018 [8]. In 2020 and 2021, the group of researchers involved in the GMp decided to postpone another edition of the report for sanitary reasons derived from the COVID-19 pandemic, and only in 2022 was it possible to release a new report.

Besides guiding policies to promote physical activity for the pediatric population, this monitoring allows for the comparability of the findings with other countries that carry out similar procedures [4,5,6]. Brazil’s Report Card project differs from other monitoring projects developed in Brazil because it not only monitors and summarizes the physical activity levels of children and adolescents but also includes other indicators related to physical activity (Behaviors, Sources of Influence, and Government Strategies and Investments) [4,5,6,7,8], which, if improved over time, will result in more active and healthier children and young people throughout life [4,5,6]. Thus, this article summarizes the 2022 results of the Report Card on Physical Activity for Brazilian children and adolescents.

## 2. Materials and Methods

The Report Card Brazil 2022 was developed based on the Canadian Report Card template [9,10,11], using a harmonized process for data collection, assessment, and the assignment of grades to indicators. For over a decade, the Canadian Report Card has been successful in raising awareness and influencing policies for physical activity promotion in childhood. Canada was the first country to develop a Report Card on physical activity in children and adolescents. Based on this report, Canadian researchers created the GMp, in which all invited countries had to adopt the Canadian methodology. The AHKGA led to the harmonized development of country-specific physical activity Report Cards, synthesizing the best available evidence on how a nation has done in terms of promoting physical activity among children and youth since 2014 [4,5,6]. The countries involved in the Report Card initiative have graded common indicators related to physical activity in children and youth using a common grading rubric and standardized benchmarks [4,5,6].

In the Report Card Brazil 2022, a group of experts led by 10 professors with doctoral degrees (all of them are authors of this article) gathered the best possible evidence on physical activity indicators in children and adolescents in Brazil. The higher education institutions that developed this process were: (1) The State University of Santa Catarina, Brazil (coordinating institution); (2) The Catholic University of Brasilia, Brazil; (3) The Universidad Catolica del Maule, Chile; (4) The State University of Santa Catarina, Brazil; (5) The University of Santiago de Chile; (6) The São Paulo State University, Brazil; (7) The Federal University of Alagoas, Brazil; (8) The State University of Maringá, Brazil; (9) The Federal University of Sergipe, Brazil; and (10) The Federal Institute of Ceará, Brazil.

All 10 experts are Full Professors in Graduate Programs, which demonstrates their responsibility in gathering the best possible evidence on the topics discussed here. Furthermore, the involvement of experts from different regions of Brazil allowed for the collective construction of the entire process and a look that is consistent with the different Brazilian realities. Each of the 10 experts involved their own research groups, which are composed of other professors and undergraduate and graduate students from the aforementioned universities and institutes. In total, approximately 80 researchers were involved in the process of gathering the best evidence for Brazil.

As was performed in previous editions of the Report Card Brazil [7,8], this edition also carried out an exhaustive but necessary process of gathering evidence for all of the indicators investigated. Ten key indicators were investigated in the GMp [4,5,6] (i.e., Overall Physical Activity, Organized Sport and Physical Activity, Active Play, Active Transportation, Sedentary Behaviors, Physical Fitness, Family and Peers, School, Community and Environment, and Government). Additionally, this was the first version of the Report Card Brazil to analyze three indicators that are directly related to physical activity (i.e., Sleep, Obesity, Poor Mental Health) [3]. Table 1 shows the indicator and benchmark used in the study.

The protocol for this research was published on the Open Science Framework (OSF) platform (https://osf.io/sjgv9, accessed on 1 March 2022). To gather the best evidence available in Brazil on each indicator, the following strategies were adopted:

(a) Fourteen systematic reviews were conducted [12,13,14,15,16,17,18,19,20,21,22,23,24,25], covering the following indicators: (1) Overall Physical Activity; (2) Organized Sport and Physical Activity; (3) Active Play; (4) Active Transportation; (5) Sedentary Behaviors; (6) Sleep; (7) Family and Peers; (8) Community and Environment; (9) Physical Fitness; (10) Obesity; (11) Poor Mental Health. In all systematic reviews, only studies with a sample of Brazilian children and adolescents published until December 2019—that is, before the COVID-19 pandemic—were considered eligible. This strategy was adopted because, in 2020 and 2021, the COVID-19 pandemic devastated Brazil and changed the dynamics of the Brazilian population, including scientific research. Thus, studies published until 2019 were considered eligible in the current edition of the Report Card Brazil. The target age for the samples investigated in the systematic reviews of this current report was adolescent children aged 5 to 19 years. All details about the samples can be consulted in previously published articles [12,13,14,15,16,17,18,19,20,21,22,23,24,25];
(b) For the School indicator, an original study [26] was conducted with public data from the National Institute for Educational Studies and Research Anísio Teixeira (INEP);
(c) For the indicator related to Governmental Strategies and Investments, analyses were based on official information from the Brazilian Federal Government, in which official websites of the different Ministries in Brazil, national surveys of the federal government, and technical reports on the subject were researched. One paper about this indicator has been published [27].

Furthermore, in all articles, the results are discussed in depth, and improvement strategies for each indicator are presented. The articles were published in peer-reviewed and open access journals [12,13,14,15,16,17,18,19,20,21,22,23,24,25,26,27].

After gathering the evidence, the group of experts met to assign grades for the indicators based on the grading framework established by AHKGA, which is used in all countries that are part of the GMp (Table 2) [4,5,6]. Briefly, the grades of each of the 10 key indicators range from A (we are succeeding with 80% of children and adolescents) to F (succeeding with <20%). Table 2 shows the description for each of the grades awarded. For indicators with incomplete, inadequate, or insufficient data, the INC grade is assigned. The Sleep indicator is not established as one of the 10 key AHKGA indicators; however, as it was an indicator investigated in the Report Card Brazil 2022, the group of experts assigned the same AHKGA grading framework for this indicator. For the Obesity and Poor Mental Health indicators, the prevalence was presented without assigning a grade. To assign a grade for the Government indicator, the instrument proposed by Bull et al. [28] and used by AHKGA was used [29].

At the consensus meeting, the group of experts discussed the results and defined the theme of Brazil’s Report Card based on the current dynamics of Brazilian society, the grades, and the priorities for each indicator for the coming years in Brazil.

## 3. Results

The monitoring results are summarized in Table 3. The best rated indicator in Brazil was School (grade B), and the indicator with the lowest grade was Active Play (grade F). The other indicators presented moderate to weak performance (grades C and D). Regarding health outcomes, 11.7% of Brazilian children and adolescents were obese, and 37.8% had poor mental health. The physical fitness indicator was classified as D+, which corresponds to a percentage from 34% to 39% of children and adolescents who meet the health criteria for different indicators of fitness.

**Figure 1:**
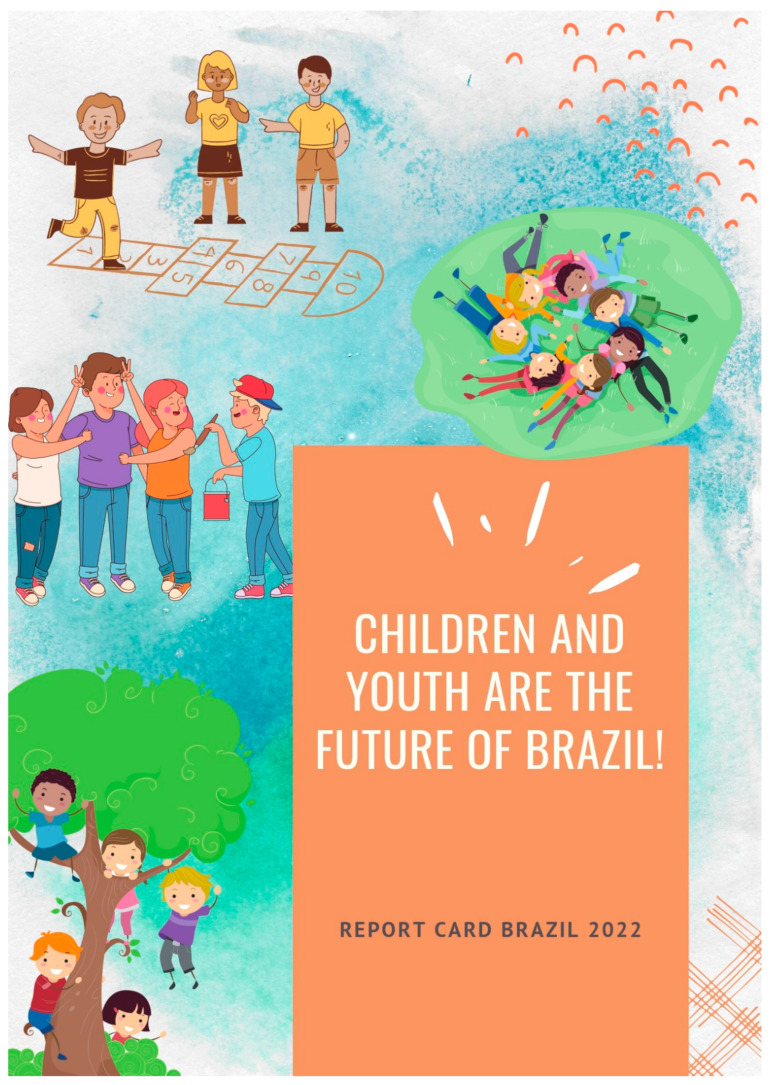
Front cover of the Report Card Brazil 2022.

Figure 1 shows the front cover of the Report Card Brazil 2022 for children and adolescents. The theme chosen for the year 2022 was “Children and youth are the future of Brazil”. The full version of the Brazil report can be consulted free of charge at https://www.activehealthykids.org/brazil/ (accessed on 1 August 2022).

## 4. Discussion

Brazil’s performance in the indicators investigated in the present study was, for the most part, from weak to moderate, drawing attention to the need to develop action plans to promote physical activity in Brazilian children and adolescents. The “Physical Activity Guidelines for the Brazilian population” [1] focused, through examples and accessible language, on strategies that can be adopted by families, schools, and managers to provide opportunities for physical activity practices in and out of school. Therefore, the results of this article, aligned with strategies of the “Physical Activity Guidelines for the Brazilian population”, can guide decision making to promote physical activity in this target population in different environmental settings.

In previous versions of the Report Card Brazil on the physical activity of children and adolescents, the results varied between C and D grades for all indicators [7,8]. The School indicator was the best rated in 2022 (grade B), and one of the reasons for this is the fact that School Physical Education (PE) is a compulsory subject in Brazilian elementary and high schools, so more than 80% of schools sampled in the School Health Survey (PENSE-Brazil) [30] reported that students had PE classes. In addition, a criterion for this indicator is the structure of schools in relation to the presence of places for the practice of sports and physical activities, such as yards, courts, and spaces intended for this purpose [4,5,6]. It is noteworthy that the quality of structures in schools for the practice of physical activity needs to be further investigated, as the potential for the improvement of this indicator is visible, mainly in some geographic regions of the country. Although PE classes are mandatory in Brazil, this is not true for all basic education (only elementary and high schools), and the number of classes (at least three per week) recommended by the Physical Activity Brazilian Guide is also not guaranteed in all Brazilian schools [1].

Furthermore, for one of the markers that compose the School indicator, Brazil does not have information/evidence (i.e., % of parents/guardians who reported that children and adolescents have access to physical activity opportunities at school, in addition to PE classes). This lack of information makes it difficult to compare with other countries, especially high-income ones, such as Canada [31] and Denmark [32], which have detailed information on the criteria evaluated in the GMp.

The indicators with the worst performances in the evaluation of the Report Card Brazil 2022 were Active Play (grade F), Overall Physical Activity (grade D), and Sedentary Behaviors (grade D), even with research showing that having sufficient levels of physical activity and reduced sedentary behavior is beneficial for physical and mental health [1,2,3]. One of the strategies to increase the level of physical activity among children and adolescents is precisely through unstructured and playful activities (i.e., Active Play) [33]. Some methodological aspects of the researched evidence need to be highlighted for these behaviors; a total of 154 publications (based on 104 different studies) were considered for the Overall Physical Activity indicator, but only 9 studies estimated the prevalence of compliance with Physical Activity recommendations using device-based measures (3 used pedometers and 6 used accelerometers) [13]. If the results were device-based measures, Brazil would have a grade of D+ (34.1% of children and young people met the recommendations), that is, a better performance than grade D (29.9% of children and young people met the recommendations) [13]. Another interesting aspect is that only 3 out of 10 Brazilian children and adolescents spend up to two hours a day in front of a screen, and most of the findings use subjective measures [12]. For Active Play, six studies that were restricted to analyzing the fact of playing or not playing did not specify the time spent on activities [21,22,23,24,25,26,27,28,29,30,31,32,33,34], which limits the proposition of strategies for guiding educators and stakeholders.

The influence of family and peers for practicing physical activities was considered moderate (C−) in our study, and it was higher in countries such as as Denmark (B−), Finland (B−), Belgium (C+), and Slovenia (B+) [35]. The data from Report Card Poland 2022 showed a similar grade to ours (C−) [36]. In all these countries, there is a concern for recommending social support as one of the strategies to promote physical activity for children and adolescents [35,36], approaching different strategies (e.g., model, incentive, logistical support, co-participation, and observation/supervision) [37].

The Community and Environment indicator was classified as a having moderate quality in Brazil (grade C). High-income countries such as Belgium, Denmark, Finland, and Sweden showed better results than Brazil [36]. A previous study has shown that children are more likely to be physically active if the neighborhood has facilities and equipment such as courts, walking and cycling lanes, playgrounds, and recreation centers, as well safety [38]. Thus, investments in environmental infrastructure, urban mobility, and safety should be part of the health policy agenda.

This is the second edition of the Report Card Brazil that monitors obesity as a health outcome. In 2018, from a systematic review [39] with a sample of Brazilian children and adolescents, 61 studies were found, and it was possible to estimate that 11.6% of Brazilian children and adolescents were obese. In the current report, an update of the previous systematic review [19] was carried out, and this estimate was 11.0%, which demonstrates that this risk factor was not modified in the last two years. Among the obesogenic factors, environmental and individual determinants that were investigated in the systematic review [19] of this report stood out.

The process of developing this report involved researchers from 10 higher education institutions and followed the procedure established by AHKGA for gathering the best evidence on all indicators under study. This careful procedure was also followed to choose the theme for the Report Card Brazil, which had the consensus of all researchers involved in the project. The choice of the theme took into account the current issue related to the COVID-19 pandemic that devastated (and continues to devastate) the country and the fact that social inequalities in Brazil have increased since 2018 and potentiate the effects of the health crisis [40,41]. Looking at future generations and envisioning better days is the role of the entire Brazilian society. In this way, the country needs to understand that: “Children and youth are the future of Brazil”, and, therefore, special attention should be given to this population.

This research has limitations that need to be highlighted. First, there is the fact that, in the research team, there is no Brazilian institution and researcher from the northern region of the country. As Brazil is a country with continental dimensions and five geographic regions (Midwestern, Northeastern, Northern, Southeastern, and Southern), cultural differences between regions are evident, and a specific look at each region is important to echo the results countrywide. Second, this project does not rely on data from research developed during the COVID-19 pandemic for this edition, which tends to change behaviors and health outcomes. Third, studies that integrated the country’s best evidence for daily behaviors had self-reported information.

The strengths of this current version of the Report Card Brazil include the fact that, for all indicators under study, the methodological details were previously described in scientific articles, and, for most of them, the systematic literature review strategy was used. In addition, the Brazil team strictly followed all AHKGA recommendations for the GMp, which demonstrates that the country is contributing to the monitoring of physical activity indicators of children and adolescents in a standardized way with other countries. Finally, Brazil has a rich collection of studies on different health indicators in children and adolescents, which can be visualized by assigning grades to all indicators of this project. This highlights the scientific development and academic quality of teaching at institutions in the country.

## 5. Conclusions

Brazil’s performance in the indicators investigated was, for the most part, from weak to moderate, which demonstrates that Brazil needs to prioritize the health and physical activity promotion of children and adolescents. Although the School indicator was well rated, the other indicators related to physical activity (Family and Peers, Community and Environment, and Government Strategies and Investments) had lower performances, which results in poor physical activity and sedentary behavior markers (Overall Physical Activity, Organized Sport and Physical Activity, Active Play, and Active Commuting). In addition, poor performances on health outcomes such as Physical Fitness, Obesity, Sleep, and Poor Mental Health were also found.

## Figures and Tables

**Table 1:** Investigated indicator and benchmark used for the development of the Report Card Brazil 2022.

Indicator	Benchmark
*Behaviors*	
Overall Physical Activity	% of children and youth who meet the Global Recommendations on Physical Activity for Health, which recommend that children and youth accumulate at least 60 min of moderate-to-vigorous-intensity physical activity per day, on average.
Organized Sport and Physical Activity	% of children and youth who participate in organized sport and/or physical activity programs.
Active Play	% of children and youth who engage in unstructured/unorganized active play at any intensity.
Active Transportation	% of children and youth who use active transportation to get to and from places (e.g., school, park, mall, friend’s house).
Sedentary Behaviors	% of children and youth who meet the Canadian Sedentary Behaviour Guidelines (no more than 2 h of recreational screen time per day).
Sleep	% of children and youth who meet the Canadian 24 h Movement Guidelines for Children and Youth.
*Sources of influence*	
Family and Peers	% of parents who meet the Global Recommendations on Physical Activity for Health, which recommend that adults accumulate at least 150 min of moderate-intensity aerobic physical activity throughout the week or do at least 75 min of vigorous-intensity aerobic physical activity throughout the week or an equivalent combination of moderate- and vigorous-intensity physical activity.
School	% of schools with active school policies (e.g., daily physical education (PE), daily physical activity, recess, “everyone plays” approach, bike racks at school, traffic calming on school property, outdoor time); % of schools where the majority (≥80%) of students are taught by a PE specialist; % of schools where the majority (≥80%) of students are offered the mandated amount of PE (for the given state/territory/region/country); % of schools that offer physical activity opportunities (excluding PE) to the majority (>80%) of their students; % of schools with students who have regular access to facilities and equipment that support physical activity.
Community and Environment	% of children or parents who perceive their community/municipality as doing a good job at promoting physical activity; % of children or parents who report having facilities, programs, parks, and playgrounds available to them in their community; % of children or parents who report living in a safe neighborhood where they can be physically active; % of children or parents who report having well-maintained facilities, parks, and playgrounds in their community that are safe to use.
*Government Strategies and Investments*	
Government	Evidence of leadership and commitment in providing physical activity opportunities for all children and youth. Allocated funds and resources for the implementation of physical activity promotion strategies and initiatives for all children and youth. Demonstrated progress through the key stages of public policymaking (i.e., policy agenda, policy formation, policy implementation, policy evaluation, and decisions about the future).
*Health outcomes*	
Physical Fitness	Average percentile achieved on physical fitness indicators
Obesity	% of obesity.
Poor Mental Health	% of mental health indicators.

**Table 2:** Methodological strategy adopted by the Global Matrix—AHKGA project to assign grades to indicators [4,5,6].

Interpretation	Grade	Prevalence (%)
Brazil is succeeding with the vast majority of children and adolescents	A+	94–100%
	A	87–93%
	A−	80–86%
Brazil is succeeding with more than half of children and adolescents	B+	74–79%
	B	67–73%
	B−	60–66%
Brazil is succeeding with about half of children and adolescents	C+	54–59%
	C	47–53%
	C−	40–46%
Brazil is succeeding with less than half of children and adolescents	D+	34–39%
	D	27–33%
	D−	20–26%
Brazil is succeeding with few children and adolescents	F	<20%
Incomplete or insufficient data for grade assignment	INC	

**Table 3:** Grades according to Physical Activity Indicator in the Report Card Brazil 2022.

Indicators	Grade
*Behaviors*	
Overall Physical Activity	D
Organized Sport and Physical Activity	C−
Active Play	F
Active Transportation	C
Sedentary Behaviors	D
Sleep	C
*Sources of influence*	
Family and Peers	C−
School	B
Community and Environment	C
*Government Strategies and Investments*	
Government	D+
*Health outcomes*	
Physical Fitness	D+
Obesity	11.7%
Poor Mental Heath	37.8%

## Data Availability

Not applicable.
